# The taste of ribonucleosides: Novel macronutrients essential for larval growth are sensed by *Drosophila* gustatory receptor proteins

**DOI:** 10.1371/journal.pbio.2005570

**Published:** 2018-08-07

**Authors:** Dushyant Mishra, Natasha Thorne, Chika Miyamoto, Christopher Jagge, Hubert Amrein

**Affiliations:** 1 Texas A&M Health Science Center, Department of Molecular and Cellular Medicine, College of Medicine, Texas A&M University, College Station, Texas, United States of America; 2 Center for Devices and Radiological Health, Food and Drug Administration, Silver Spring, Maryland, United States of America; University of Lausanne, Switzerland

## Abstract

Animals employ various types of taste receptors to identify and discriminate between different nutritious food chemicals. These macronutrients are thought to fall into 3 major groups: carbohydrates/sugars, proteins/amino acids, and fats. Here, we report that *Drosophila* larvae exhibit a novel appetitive feeding behavior towards ribose, ribonucleosides, and RNA. We identified members of the gustatory receptor (Gr) subfamily 28 (Gr28), expressed in both external and internal chemosensory neurons as molecular receptors necessary for cellular and appetitive behavioral responses to ribonucleosides and RNA. Specifically, behavioral preference assays show that larvae are strongly attracted to ribose- or RNA-containing agarose in a *Gr28*-dependent manner. Moreover, Ca^2+^ imaging experiments reveal that *Gr28a*-expressing taste neurons are activated by ribose, RNA and some ribonucleosides and that these responses can be conveyed to *Gr43a*^*GAL4*^ fructose-sensing neurons by expressing single members of the *Gr28* gene family. Lastly, we establish a critical role in behavioral fitness for the *Gr28* genes by showing that *Gr28* mutant larvae exhibit low survival rates when challenged to find ribonucleosides in food. Together, our work identifies a novel taste modality dedicated to the detection of RNA and ribonucleosides, nutrients that are essential for survival during the accelerated growth phase of *Drosophila* larvae.

## Author summary

Insects that undergo complete metamorphosis grow only during the larval stage of development. In many species, this period is restricted to a few days, during which larvae might increase their weight up to several hundred-fold. *Drosophila melanogaster*, for example, grow from a tiny first-instar larva of about 10 μg to a wandering third-instar larva weighing about 2 mg over a period of only 4.5 days. The main macronutrients known to be critical for this period of rapid growth are amino acids and sugars. In this study, we identify ribonucleosides and RNA as a new, additional type of nutrient necessary for rapid larval growth and survival. We show that larvae harbor taste neurons that express taste receptors necessary for sensing ribonucleosides and RNA. Larvae lacking these taste receptors show high mortality rates when exposed to a complex food environment that requires the location of ribonucleoside-containing food. We hypothesize that the ability to taste RNA evolved as a new taste modality in larvae of insects that go through a rapid growth period because ingestion of ribonucleosides, as opposed to de novo synthesis, provides a survival advantage during a period of extreme growth.

## Introduction

Taste discrimination is a common trait of all animals, the most crucial being the ability to distinguish between palatable and mostly nutritional chemicals from aversively perceived, often harmful, and generally bitter-tasting compounds. Calorically nutritious food compounds fall into 3 categories, fats, proteins, and carbohydrates, and their consumption is dependent not only on availability but also on internal physiological states of the animal, such as overall nutrition status, anticipated need for energy expenditure, developmental stage, and reproductive status. To achieve discrimination between different nutrients, different subsets of cells in the taste sensory system express specific receptors for the detection of chemicals belonging to these nutrient groups [1].

Growth of most arthropods, but also some vertebrates, is characterized by a period of rapid body weight gain within a few days. A mouse increases its weight from time of birth (approximately 1 g) to the time of weaning (about 3 weeks; about 10 to 13 g) by about a factor of 10. Growth dynamics of many insect larvae are even more dramatic. A freshly hatched *Drosophila* larva weighs about 9.5 μg [2] but grows over a period of only 108 hours to more than 1.5 mg at the time of puparium formation [3], which translates to doubling of weight about every 14 hours. To support this rapid growth, animals are required to consume large amounts of all essential macronutrients, especially carbohydrates, proteins, and fats. In the natural diet of *Drosophila*, fruits provide the carbohydrates, while fatty acid and proteins are obtained mostly from colonizing microorganisms, such as yeast. Standard *Drosophila* food used in most laboratories is generally composed of cornmeal, malt extract, and yeast extracts (see Material and methods), which contain all these and other macro- and micronutrients in abundance.

In contrast to flies, which express and employ 8 sugar *Gr* genes for the perception of sweet taste, larvae use a single sugar receptor, Gr43a, for the detection of fructose and sucrose, the most abundant sugars in most fruits [4]. In addition, an Ionotropic Receptor (IR) was recently shown to be involved in sensing a limited number of amino acids [5]. Receptors for other macronutrients—including many amino acids, fats, and other compounds potentially critically important for rapid larval growth—have not been identified to date.

Here, we report the discovery of a novel taste modality—the taste of ribonucleosides and RNA—and the identification of cognate receptors. We show that larvae have a strong attraction to and feeding preference for ribose, ribonucleosides, and RNA. Feeding on these molecules is mediated by members of the highly conserved taste receptor subfamily Gr28. Live imaging experiments using the Ca^2+^ sensor Calcium Modulated Photoactivatable Ratiometric Integrator (CaMPARI) show that *Gr28a-GAL4*-expressing taste neurons respond to ribose, inosine, and uridine, as well as RNA itself, but not to 2-deoxyribose or to adenosine, guanosine, or cytidine. Holidic (synthetic) medium (HM) lacking inosine and uridine slows larval growth and causes high mortality, and supplementing this medium with RNA rescues both phenotypes. Moreover, when provided with a choice of HM with and without inosine and uridine, wild-type larvae readily select complete HM, leading to fast growth and high survival, while *Gr28* mutant larvae fail to do so, resulting in slow growth and low survival. In summary, we have identified the cellular and molecular basis for the taste of ribonucleosides and RNA. We suggest that *Drosophila* larvae—and possibly other insect larvae—need to feed on RNA precursors to sustain the rapid increase in body weight, which is doubled almost twice a day.

## Results

### Ribose and RNA precursors elicit appetitive taste responses in *Drosophila* larvae

**Fig 1:**
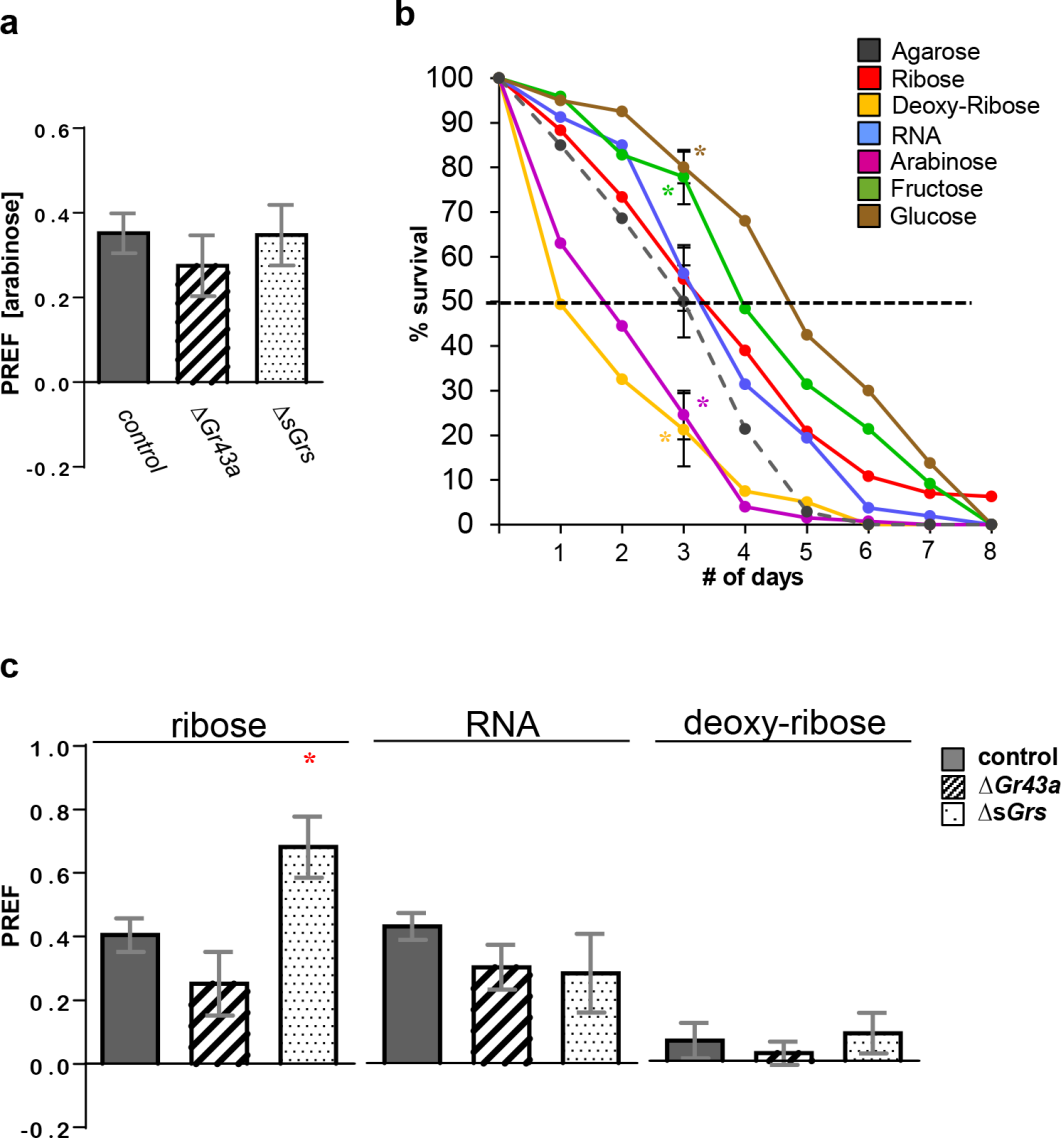
Larval preference for ribose and RNA is not mediated by sugar *Gr* genes. **Two-choice preference assays for arabinose, ribose, deoxyribose, and RNA (panel a and c) and survival on these chemicals and nutritious sugars (panel b).** (a) Preference for arabinose is independent on various sugar *Gr* genes (*n* = 12–28). The underlying data can be found in S1 Data. (b) Comparison of survival of *w*^*1118*^ larvae when kept on different substrates (*n* = 3–8). After 72 hours, approximately 50% of the larvae survive on agarose-only substrate (median survival, dashed line). For simplicity, significant differences are only indicated for median survival time. Data are represented as mean ± SEM. “*” represents significant difference between the larval survival on different substrates and agarose (two-tailed Mann-Whitney U test, *p* < 0.05). Reduced survival rate of larvae kept on arabinose and deoxyribose might be due to interference of these chemicals with sugar metabolism. The underlying data can be found in S2 Data. (c) Larvae show strong preference for ribose (n = 12–36) and RNA (n = 6–36) when lacking *Gr43a* or the 8 *sGr*. Larvae are not attracted to deoxyribose (n = 6–24). As for fructose [4], Δ*sGr* larvae showed stronger preference for ribose than wild-type larvae. Concentration of all substrates was 100 mM in 1% agarose, except RNA (0.5 mg/mL in 1% agarose). Genotypes: *w*^*1118*^ (control), *w*^*1118*^*; Gr43a*^*GAL4*^*/Gr43a*^*GAL 4*^*(*Δ*Gr43a*), and *w*^*1118*^; Δ*Gr61a* Δ*Gr64a-f/*Δ*Gr61a* Δ*Gr64a-f (*Δ*sGrs*). The underlying data can be found in S1 Data. Gr, gustatory receptor; PREF, preference index; *sGr*, sugar *Gr* gene.

While assessing sugar specificity of Gr43a, we made the surprising observation that larvae are also strongly attracted to arabinose (Fig 1). When given the choice between agarose containing L-arabinose and agarose alone, larvae showed a strong preference for arabinose (Fig 1A). In flies, arabinose is detected by receptors encoded by the sugar *Gr* subfamily [6, 7], none of which are expressed in larvae [8, 9]. Instead, larvae sense the main fruit sugars sucrose and fructose through Gr43a, which is expressed in pharyngeal taste neurons, as well as nutrient-sensing neurons in the brain [8]. Other nutritious sugars, such as trehalose and glucose, are sensed postprandially by Gr43a-expressing neurons in the brain, presumably after the conversion of a fraction of these sugars into fructose [8, 10]. To examine the possibility that arabinose is sensed either through Gr43a or any of the main sugar receptors used by adult flies, the low expression of which might have been missed in previous studies, we examined arabinose preference in *Gr43a* mutant larvae or larvae lacking all 8 sugar *Gr* genes (octuple mutant strain; Fig 1A [11]). However, neither *Gr43a* mutant nor octuple mutant larvae showed any significant loss in arabinose preference in the two-choice preference assay (Fig 1A).

L-arabinose is present as a minor component in heteropolysaccharides, such as hemicellulose and pectin [12, 13], but it is not a major sugar in fruit and cannot be metabolized by flies [14, 15]. To assess whether larvae can use arabinose as an energy source, we compared survival rates of second-instar larvae kept on agarose-containing arabinose to larvae kept on plain agarose or nutritious sugar-containing agarose (Fig 1B). Median (50%) survival for larvae on plain agarose was 3 days. Larvae kept on nutritious sugar–containing agarose survived significantly better, with more than 75% of larvae still alive after 3 days, indicating that the consumed sugar provided energy and decreased mortality. In contrast, only about 30% of larvae kept on arabinose-containing agarose survived to the 3-day time point. These observations suggest that arabinose cannot be a nutritionally relevant ligand, and we posit that larvae instead detect a molecule structurally related to arabinose, but one that is nutritious and essential for larval growth.

Arabinose is closely related to ribose, the carbohydrate backbone of RNA. We therefore investigated whether ribose and RNA can elicit a similar preference. Indeed, larvae strongly preferred ribose and RNA over plain agarose in the two-choice preference assay, whereas 2-deoxyribose, the sugar moiety of DNA, did not elicit a preference (Fig 1C). Moreover, neither *Gr43a* nor the sugar Gr genes were required to sense either ribose or RNA, as larvae with respective mutations showed robust preference for both substrates (Fig 1C). We next tested how ribose- or RNA-containing agarose affected larval survival (Fig 1B). In contrast to arabinose, neither of these compounds reduced survival time. However, they did not serve as an efficient energy source either because median survival time was not significantly different from larvae kept on plain agarose (Fig 1B). Taken together, these findings suggest that larvae can sense RNA through a taste or internal chemosensory receptor, presumably recognizing the ribose moiety in the RNA backbone.

### Ribose and RNA sensing is mediated by members of the Gr28 protein family

**Fig 2:**
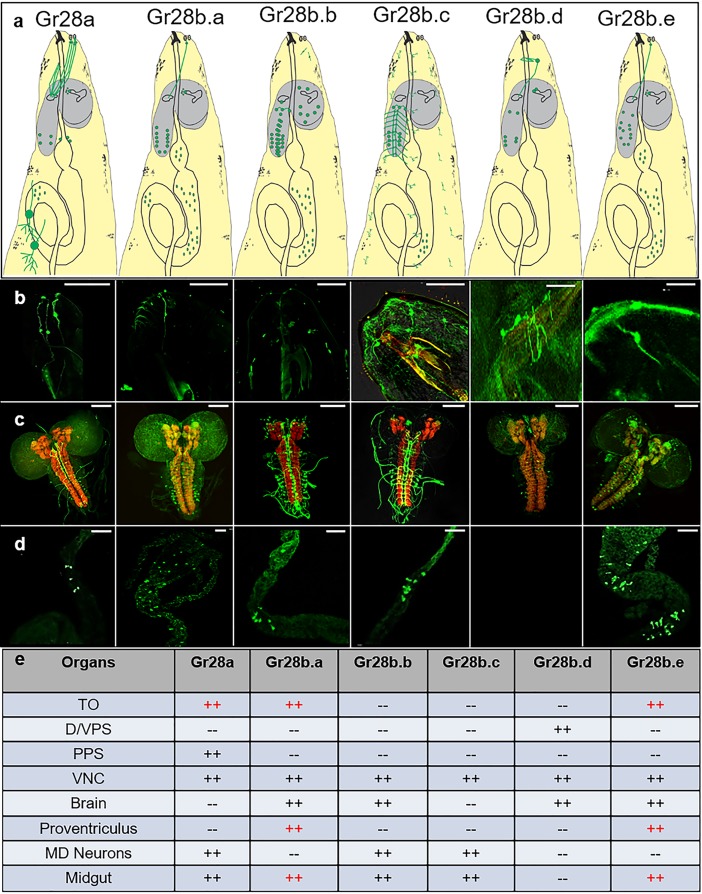
Expression of the 6 *Gr28* genes in third-instar larvae. (a) Graphic summary of *Gr28* gene expression. Cells and neurons with their axons expressing the respective GAL4 driver are all shown in green. Brain is shown in grey, and the digestive system—including the pharynx, PV, and gut—are outlined. (b) Live GFP imaging of the larval head, showing expression of 3 genes (*Gr28a*, *Gr28b.a*, and *Gr28b.e*) in neurons of the TO. *Gr28b.d* is expressed in neurons of the DPS and VPS organs, while *Gr28a* is also expressed in the PPS organ. Neither *Gr28a* nor *Gr28b.d* are co-expressed with *Gr43^GAL4^* (see S1 Fig). Number of larvae with GFP positive taste neurons/total number of larvae analyzed were 7/7 for *Gr28a*, 4/4 for *Gr28b.a*, 0/5 for *Gr28b.b*, 0/7 for *Gr28b.c*, 5/5 for *Gr28b.d*, and 5/5 for *Gr28b.e*. (c) View of the brain and parts of the ventral nerve cord, showing different degrees of expression for each of the 6 *Gr28* genes. The brains were stained with anti-GFP antibody (green) and counterstained with nc82 antibody (red). Number of larvae with GFP antibody–positive staining in the brain-VNC/number of brains analyzed were 3/3 for *Gr28a*, 5/5 for *Gr28b.a*, 3/3 for *Gr28b.b*, 5/5 for *Gr28b.c*, 3/3 for *Gr28b.d*, and 6/6 for *Gr28b.e*. (d) Live GFP imaging of the PV and midgut, showing expression of all *Gr28* genes with the exception of *Gr28b.d*. Expression of *Gr28b.a* and *Gr28b.e* is broad and includes the PV and midgut, while expression of *Gr28a* and *Gr28b.b* is defined to a smaller area of the gut only. Number of larvae with GFP-positive cells/total number of larvae analyzed were 5/5 for *Gr28a*, 4/4 for *Gr28b.a*, 3/3 for *Gr28b.b*, 5/5 for *Gr28b.c*, 0/5 for *Gr28b.d*, and 4/4 for *Gr28b.e*. (e) Summary of tissues expressing each of the 6 *Gr28* genes. Genotypes were *w*; *UAS-mCD8GFP/Gr28x-GAL4*, such that x refers to indicated *Gr-Gal4* driver. Scale bar is 100 μm. For live imaging (panel b and d), at least 5 larvae for each genotype were analyzed, and GFP cells in taste sensilla and the gut were observed in each case for *Gr28a*, *Gr28ba*, *Gr28b.e*, *Gr28b.d*, and *Gr28b.e*; for staining (panel c), at least 3 brains for each genotype were analyzed, with GFP-positive neurons observed in each case. The images are good representatives of these experiments. DPS, dorsal pharyngeal sensory; GFP, green fluorescent protein; *Gr28*, gustatory receptor subfamily 28; PPS, posterior pharyngeal sensory; PV, proventriculus; TO, terminal organ; VPS, ventral pharyngeal sensory.

We next sought to identify the bona fide receptor(s) that mediate taste preference for RNA and ribose. Most *Gr* genes expressed in larvae have not been functionally characterized during that life stage, but expression and function of many of them have been investigated in adults. The vast majority of these genes (i.e., encoding bitter taste receptors) are expressed in bitter taste neurons of taste bristles in the labial palps and legs of the fly, and it has been shown that bitter Gr proteins form multimeric receptor complexes that are activated by a vast array of chemicals perceived as repulsive [16–19]. In addition, Gr21a and Gr63a, which are also expressed in larvae, have noncanonical roles in a small subset of olfactory neurons in the fly, where they function as carbon dioxide receptors [20, 21]. Because bitter taste receptors and Gr21a/Gr63a are likely to have similar roles in larvae [9], we focused on the *Gr28* gene clade (*Gr28a*, *Gr28b*.*a*, *Gr28b*.*b*, *Gr28b*.c, *Gr28b*.*d*, and *Gr28b*.*e*), members of which show broad expression in larvae as well as adult flies [22], whose roles in chemosensation have remained enigmatic. These receptors are also highly conserved across most arthropod orders [23–25], suggesting important functions that are shared across a range of species. Some of these *Gr* genes have been implicated in ultraviolet (UV) light sensing in larvae [26], as well as temperature sensing in flies [27], but no chemical ligands for any of these Gr28 proteins have been identified. To investigate a possible role for proteins encoded by the *Gr28* gene family in RNA and ribose sensing, we first examined expression of respective GAL4 lines more closely in larvae, focusing on chemosensory organs and the digestive system, as well as the central nervous system (CNS) (Fig 2). Four of the six *Gr28* genes were expressed in small numbers of external (*Gr28a*, *Gr28b*.*a*, *Gr28b*.*e*) and pharyngeal taste (*Gr28a* and *Gr28b*.*d*) neurons. Other sites of expression included neurons in the proventriculus (*Gr28b*.*a*, *Gr28b*.*e*), cells in the gut (all but *Gr28b*.*d*), multidendritic neurons in the larval body wall (*Gr28a*, *Gr28b*.*c*, *Gr28b*.*d*), and many neurons in the CNS (*Gr28b*.*a*, *Gr28b*.*b*, *Gr28b*.*d*, and *Gr28b*.*d*). Expression of *Gr28-GAL4* drivers in the taste system did not overlap with *Gr43a*^*GAL4*^, which is expressed in a different set of pharyngeal neurons (S1 Fig).

**Fig 3:**
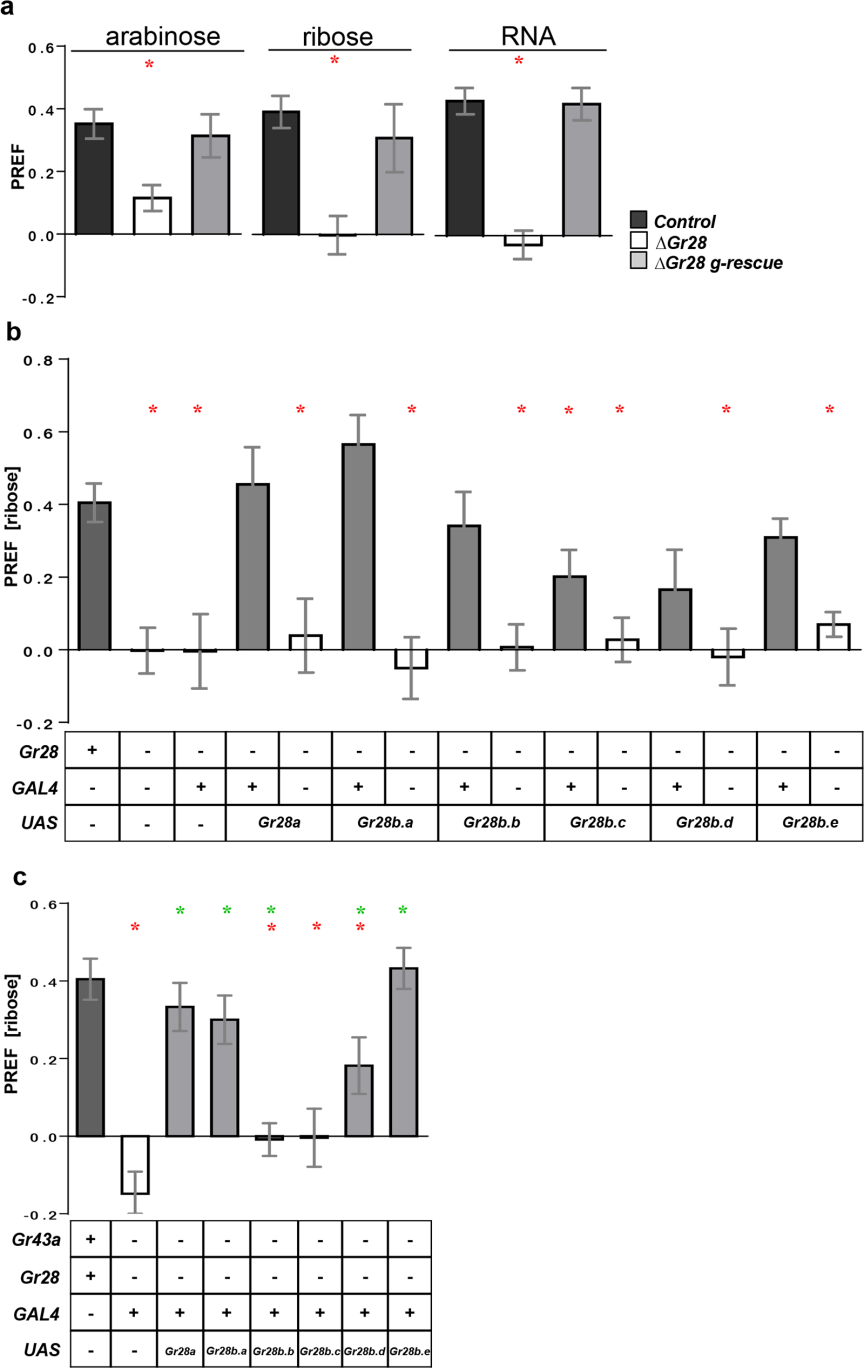
Genes of the Gr28 locus mediate larval taste preference for ribose, RNA, and arabinose. Two-choice feeding assays of wild-type and *Gr28* mutant larvae. (a) Larvae require the *Gr28* genes for taste preference for arabinose (*n =* 12–51), ribose (*n =* 12–36), and RNA (*n =* 21–36); Genotypes: *w*^*1118*^ (*Control*), *w*^*1118*^; Δ*Gr28/*Δ*Gr28* (Δ*Gr28*) *and* Δ*Gr28/*Δ*Gr28; Gr28 genomic rescue/*+ (Δ*Gr28 g-rescue*). (b) Single *Gr28* genes rescue taste preference for ribose in Δ*Gr28* homozygous mutant larvae. Genotypes were *w*^*1118*^ (lane 1), *w*^*1118*^; Δ*Gr28/*Δ*Gr28* (2), *w*^*1118*^;Δ*Gr28/*Δ*Gr28; Gr28a-GAL4/+* (3), *w*^*1118*^;Δ*Gr28/*Δ*Gr28; Gr28a-GAL4/UAS* (4, 6, 8, 10, 12, 14), and Δ*Gr28/*Δ*GrGr28; +/UAS* (5, 7, 9, 11, 13, 15) such that *UAS* represents indicated *transgene* (*n =* 12–36). (c) *Gr28* mutant larvae expressing a single *Gr28* gene in fructose-sensing (*Gr43a*^*GAL*^ -expressing) neurons show preference for ribose. Genotypes: *w*^*1118*^ (lane 1), *w*^*1118*^; Δ*Gr28 Gr43*^*GAL4*^*/*Δ*Gr28 Gr43*^*GAL4*^ (2), and *w*^*1118*^; Δ*Gr28 Gr43*^*GAL4*^*/*Δ*Gr28 Gr43*^*GAL4*^*; UAS/+*, such that *UAS* represent indicated transgene (*n =* 12–30). Each bar represents the mean ± SEM of two-choice preference responses. Concentrations were 100 mM (arabinose and ribose) or 0.5 mg/mL (RNA) in 1% agarose. Red “*” represents significant difference between indicated genotype and *w*^*1118*^ control (two-tailed Mann-Whitney U test, *p <* 0.05). Green “*” represents significant difference between indicated genotype and Δ*Gr28 Gr43a*^*GAL4*^ double mutant (*w*^*1118*^; Δ*Gr28 Gr43*^*GAL4*^*/*Δ*Gr28 Gr43*^*GAL4*^). Two-tailed Mann-Whitney U test, *p* < 0.05). The underlying data can be found in S1 Data.

We next investigated possible effects on preference for arabinose, ribose, and RNA across a range of concentrations in larvae lacking all *Gr28* genes (Δ*Gr28*; [28], Fig 3A and S2). Indeed, Δ*Gr28* mutant larvae completely lost preference for ribose and RNA and showed significantly reduced preference for arabinose, phenotypes that were rescued when reintroducing a genomic construct containing the entire *Gr28* locus (Fig 3A). These observations indicate that one or several members of the *Gr28* gene cluster mediate the detection of ribose and RNA.

To examine whether a single Gr protein can establish ribose sensing, we subjected Δ*Gr28* mutant larvae expressing each of the *Gr28* genes under the control of *Gr28a-GAL4*. All *Gr28* genes were able to restore at least some preference for ribose (Fig 3B). Notably, *Gr28a*, *Gr28b*.*a*, and *Gr28b*.*e*, all of which are expressed in taste neurons (Fig 2A), and *Gr28b*.*b*, rescued ribose preference to levels comparable to *w*^*1118*^ controls (Fig 3B). We then asked whether ribose sensing could be conveyed upon other larval taste neurons. We choose the well-characterized sugar-sensing, pharyngeal taste neurons expressing Gr43a, a receptor narrowly tuned to the sugars fructose and sucrose [8]. We examined two-choice preference behavior of Δ*Gr28* mutant larvae expressing each of the 6 Gr28 genes individually under the control of the *Gr43a*^*GAL4*^ driver (Fig 3C). Indeed, 3 genes—*Gr28a*, *Gr28b*.*a*, and *Gr28b*.*e*—endowed such larvae with the ability to sense and preferentially feed on ribose containing agarose in a manner indistinguishable from *Gr28*^*+*^ control larvae, and a fourth gene (*Gr28b*.*d*) mediated reduced ribose preference. The two other *Gr28* genes, *Gr28b*.*b* and *Gr28b*.c, failed to convey any preference, just like *Gr28* homozygous mutant larvae (Fig 3C). Of note, neither *Gr28b*.*b* nor *Gr28b*.c is expressed in taste neurons of wild-type larvae (Fig 2B), and while they rescued ribose sensing in *Gr28a* neurons, they failed to do so in heterologous *Gr43a* pharyngeal fructose-sensing neurons. One possibility is that *Gr28* taste neurons express a cofactor (i.e., a chaperone or coreceptor) absent in *Gr43a* neurons and that some, but not all, *Gr28* proteins are completely dependent on such a factor for taste receptor function. Taken together, our experiments established that larvae possess a taste modality for ribose and RNA and that individual Gr28 proteins are able to mediate ribose and RNA sensing.

### Taste neurons respond to RNA and ribose and require Gr28 proteins

**Fig 4:**
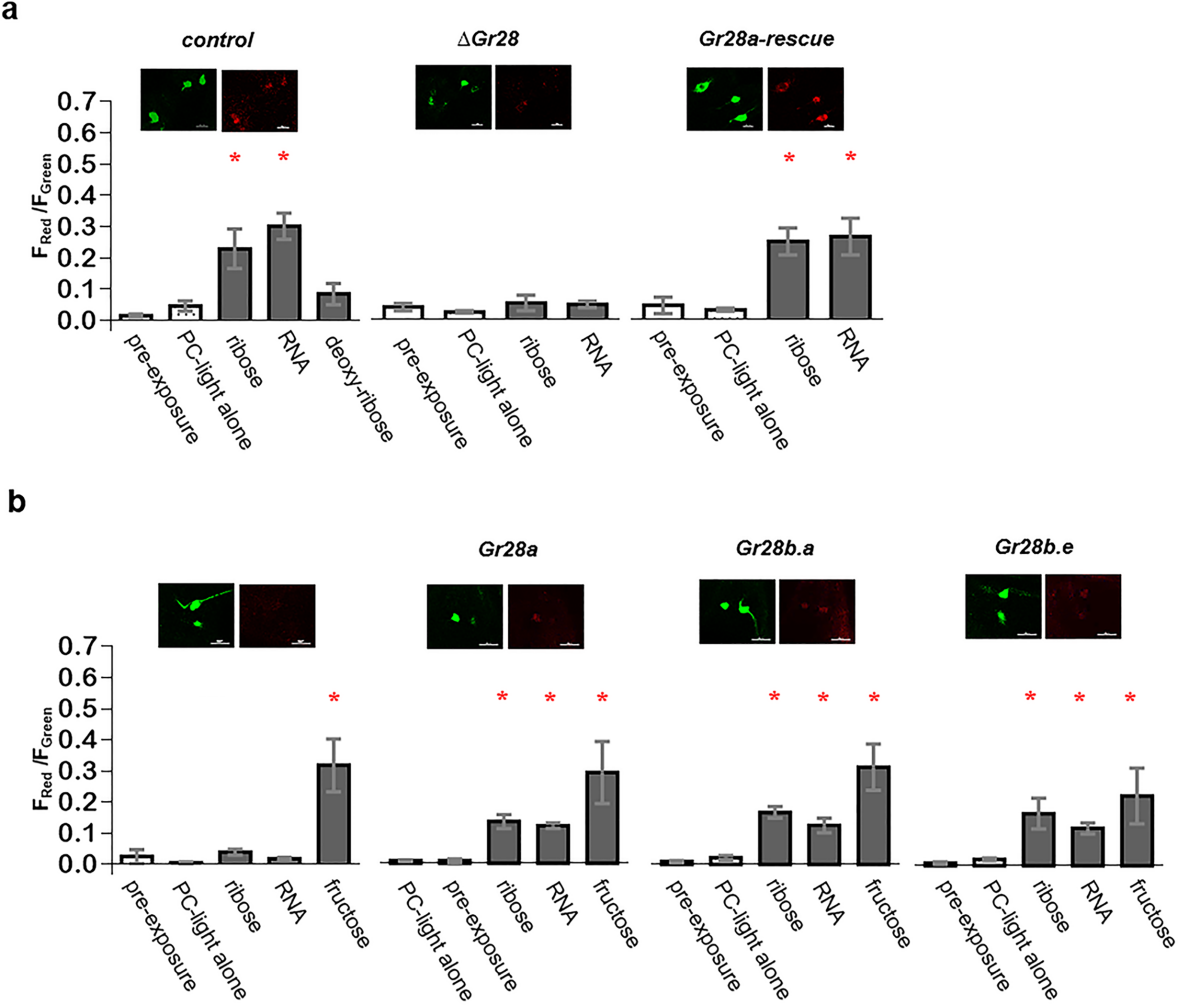
RNA and ribose are ligands for single Gr28 proteins. **(**a) Terminal taste neurons expressing the Ca^2+^ sensor CaMPARI require *Gr28a* in order to respond to ribose and RNA (*n =* 4–9). Genotypes: *w*^*1118*^*; UAS-CaMPARI/+; Gr28a-GAL4/+* (*control*), *w*^*1118*^; Δ*Gr28 UAS-CaMPARI/*Δ*Gr28; Gr28a-Gal4/+* (ΔGr28), and *w*^*1118*^; Δ*Gr28 UAS-CaMPARI /*Δ*Gr28; Gr28a-GAL4/UAS-Gr28a* (*Gr28a rescue*). (b) Expression of single *Gr28* genes conveys ribose and RNA responses to fructose-sensing pharyngeal taste neurons (*n =* 4–13). Genotypes: *w*^*1118*^*; UAS-CaMPARI Gr43a*^*GAL4*^*/+* (*control*), *w*^*1118*^*; Gr43*^*GAL4*^ *UAS-CaMPARI/+; UAS-Gr28a/+* (*Gr28a*), *w*^*1118*^*; Gr43*^*GAL4*^ *UAS-CaMPARI/+; UAS-Gr28b*.*a/+* (*Gr28b*.*a*), *w*^*1118*^*; Gr43*^*GAL4*^ *UAS-CaMPARI/+; UAS-Gr28b*.*e/+* (*Gr28b*.*e*). Final concentration of all substrates was 100 mM in water except for RNA (0.5 mg/mL). Representative images of the indicated genotypes are shown above the graphs. Scale bar is 10 μm. Each bar represents the mean ± SEM of ratios of red and green fluorescence intensities. “*” represents significant differences between the preexposure (no PC light, no chemical) group and a substrate group (two-tailed Mann-Whitney U test, *p <* 0.05). The underlying data can be found in S3 Data. PC, photoconversion.

To establish a role for the Gr28 proteins in ribose detection at the cellular level, we measured responses of terminal organ (TO) taste neurons using the fluorescent Ca^2+^ sensor CaMPARI [29]. In the presence of a high concentration of Ca^2+^ and simultaneous exposure to blue light, CaMPARI undergoes an irreversible conformational change, leading to a shift in emission properties from green to red fluorescence [29]. This feature allows application of ligand under free-moving conditions of the animal, while neural activation can be analyzed subsequently by quantifying green to red conversion ratios using fluorescent microscopy (see Material and methods). We presented *Gr28a-GAL4; UAS-CaMPARI* larvae with various ligands while illuminating them with blue light and found that ribose and RNA activated *Gr28a-GAL4* neurons, while 2-deoxyribose failed to do so (Fig 4A). Consistent with behavioral assays of Δ*Gr28* mutant larvae, neurons lacking the *Gr28* locus were not activated by either RNA or ribose, phenotypes that were rescued in the presence of a *Gr28a* transgene expressed in these neurons.

To further explore the role of the Gr28 proteins and to assess whether they can mediate ribose sensing to heterologous taste neurons, we co-expressed *UAS-CaMPARI* with *Gr28* genes in fructose-sensing pharyngeal sweet taste neurons using *Gr43a*^*GAL4*^ and measured cellular responses to ribose and RNA (Fig 4B). We chose *Gr28a*, *Gr28b*.*a*, and *Gr28b*.*e* because they were competent to mediate ribose preference behaviorally when expressed in these neurons (Fig 3C). Indeed, *Gr43a*^*GAL4*^ fructose-sensing neurons now responded to ribose and RNA in the presence of any of the 3 *Gr28* genes. The fructose response in these neurons was similar to that in neurons of control flies (i.e., not expressing a *Gr28* gene), indicating that the function of the endogenous fructose receptor was not impaired. Response to RNA or ribose was lower than responses to fructose, however, suggesting that fructose is more potent in activating Gr43a than ribose or RNA is in activating the Gr28 proteins. Alternatively, activation of Gr28 proteins might be suboptimal in heterologous taste neurons due to the absence *Gr28* neuron-specific cofactors or facilitators (see above). Regardless, these experiments, along with the two-choice feeding experiments (Fig 3C), show that expression of a single Gr28 protein can convey ribose- and RNA-sensing properties to a taste neuron normally not responding to these compounds.

### Ribonucleosides are essential for growth and survival

**Fig 5:**
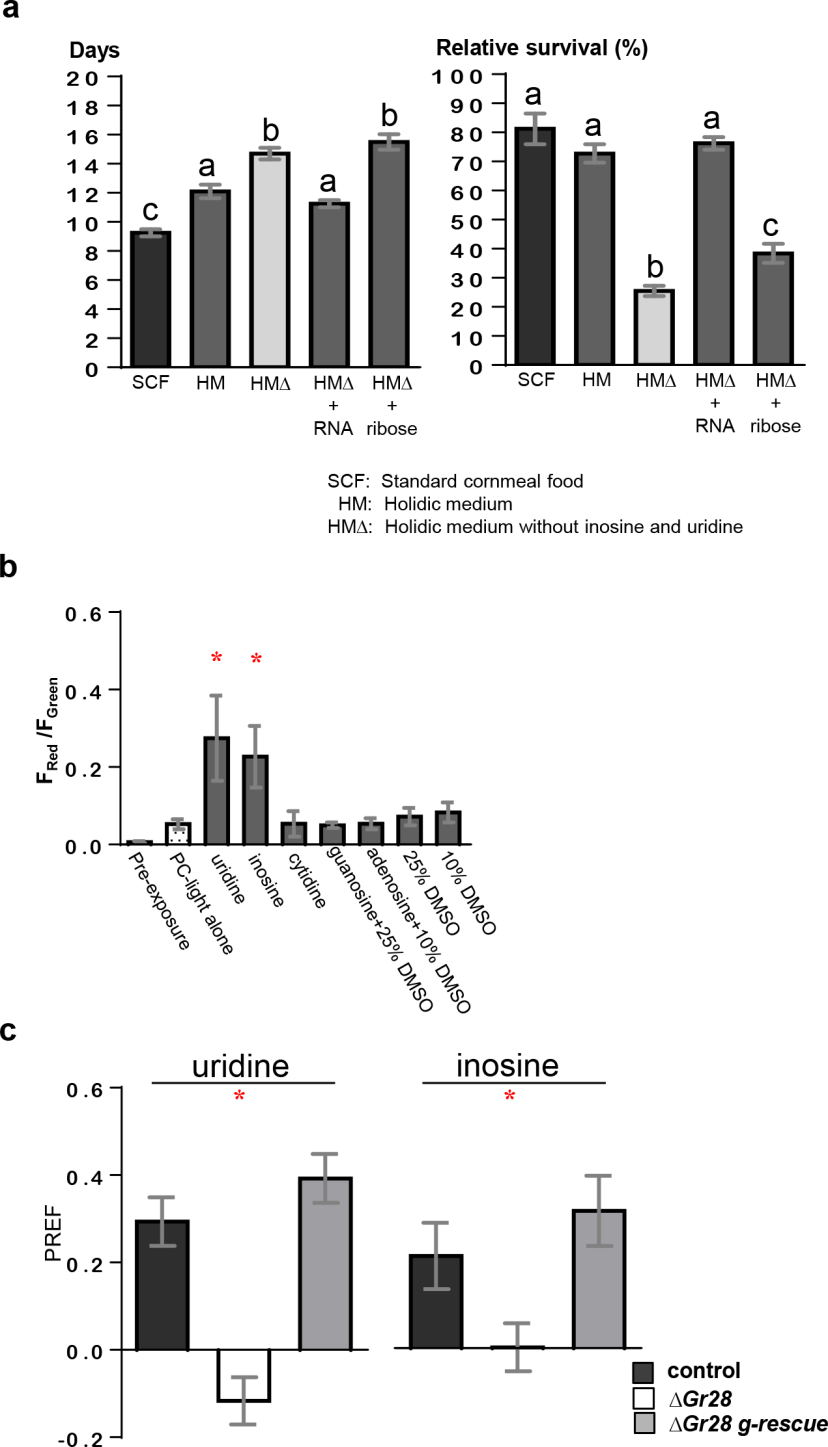
Ribonucleosides are essential nutrients for rapid larval growth and survival. (a) Growth time in days from hatching of the first-instar larvae to eclosion (left) and survival rate (right) of larvae raised in different media shows that inosine and uridine are essential components. Larvae raised on HM grow slightly slower than, but have the same survival rate as, larvae raised on SCF. Replacing ribonucleosides with RNA (0.5 mg/mL) in HMΔ restores both growth time and survival rate, while replacing it with equimolar concentration of ribose fails to do so. Each bar represents the mean ± SEM (*n* = 4). Bars with different letters represent significant differences (two-tailed Mann-Whitney U test, *p <* 0.05). Genotype: *w*^*1118*^. The underlying data can be found in S4 Data. (b) CaMPARI imaging of TO taste neurons shows that inosine and uridine, but none of the 3 other ribonucleosides, are potent ligands for Gr28 neurons. Uridine, cytidine (100 mM), and inosine (50 mM) were dissolved in water, while guanosine and adenosine were dissolved in DMSO and presented at concentration of 25 mM and 50 mM in water containing 25% and 10% f.c. DMSO, respectively. Each bar represents the mean ± SEM of ratios of red and green fluorescence intensities (*n* = 5–19). “*” represents significant differences between the preexposure (untreated) group to the groups with the indicated ligands applied (two-tailed Mann-Whitney U test, *p <* 0.05). Genotype: *w*^*1118*^*; UAS-CaMPARI/Gr28a-GAL4*. The underlying data can be found in S3 Data. (c) Two-choice preference assay shows that larvae require the *Gr28* genes to exhibit preference for uridine (50 mM, *n =* 12–24) and inosine (100 mM, *n* = 12–18). “*” represents significant difference between the genotypes (two-tailed Mann-Whitney U test, *p <* 0.05). All the genotypes are compared to control. Genotypes: *w*^*1118*^, *w*^*1118*^; Δ*Gr28/*Δ*Gr28 and w*^*1118*^; Δ*Gr28/*Δ*Gr28; genGr28/+*. The underlying data can be found in S1 Data. f.c., final concentration; HM, holidic medium; SCF, standard cornmeal food; TO, terminal organ.

In contrast to fructose or sucrose, ribose does not act as a major energy source (Fig 1B). Thus, we considered roles of RNA and its precursors as essential nutrients for larval growth. Inosine and uridine are components of HM, a synthetic *Drosophila* food medium composed of approximately 40 pure chemicals that can sustain larval growth, adult development and fertility [30]. To examine the contribution of these compounds for growth and viability, we examined developmental progression and survival rate of larvae kept on HM, HM lacking inosine and uridine (HMΔ), and modified HM in which RNA was added back to HMΔ (HMΔ + RNA; Fig 5A; see Material and methods). Confirming the findings of Piper and colleagues, HM supported larval growth and adult development with the same survival rate as larvae kept on standard cornmeal food (SCF), albeit at a slightly reduced pace. In contrast, lack of ribonucleosides in the medium (HMΔ) sharply increased mortality rate and significantly extended the larval growth phase, while adding back RNA (HMΔ + RNA)—but not ribose (HMΔ + ribose)—restored both survival and developmental time. Thus, RNA or the RNA precursors uridine and inosine are essential for rapid growth and viability. Given that ribonucleosides contain a ribose moiety, we expected that they are ligands for the Gr28 proteins and could activate TO taste neurons (Fig 5B). To test this, we first performed CaMPARI imaging experiments of wild-type larvae and found that uridine and inosine activated *Gr28a-GAL4* neurons, but the other 3 ribonucleosides (guanosine, cytidine and adenosine) did not (Fig 5B). Second, we subjected wild-type and Δ*Gr28* mutant larvae to the two-choice preference assay and found that larvae were attracted to uridine and inosine in a *Gr28-*dependent manner (Fig 5C).

### Gr28 proteins are required for ribonucleoside sensing in a challenging food environment

**Fig 6:**
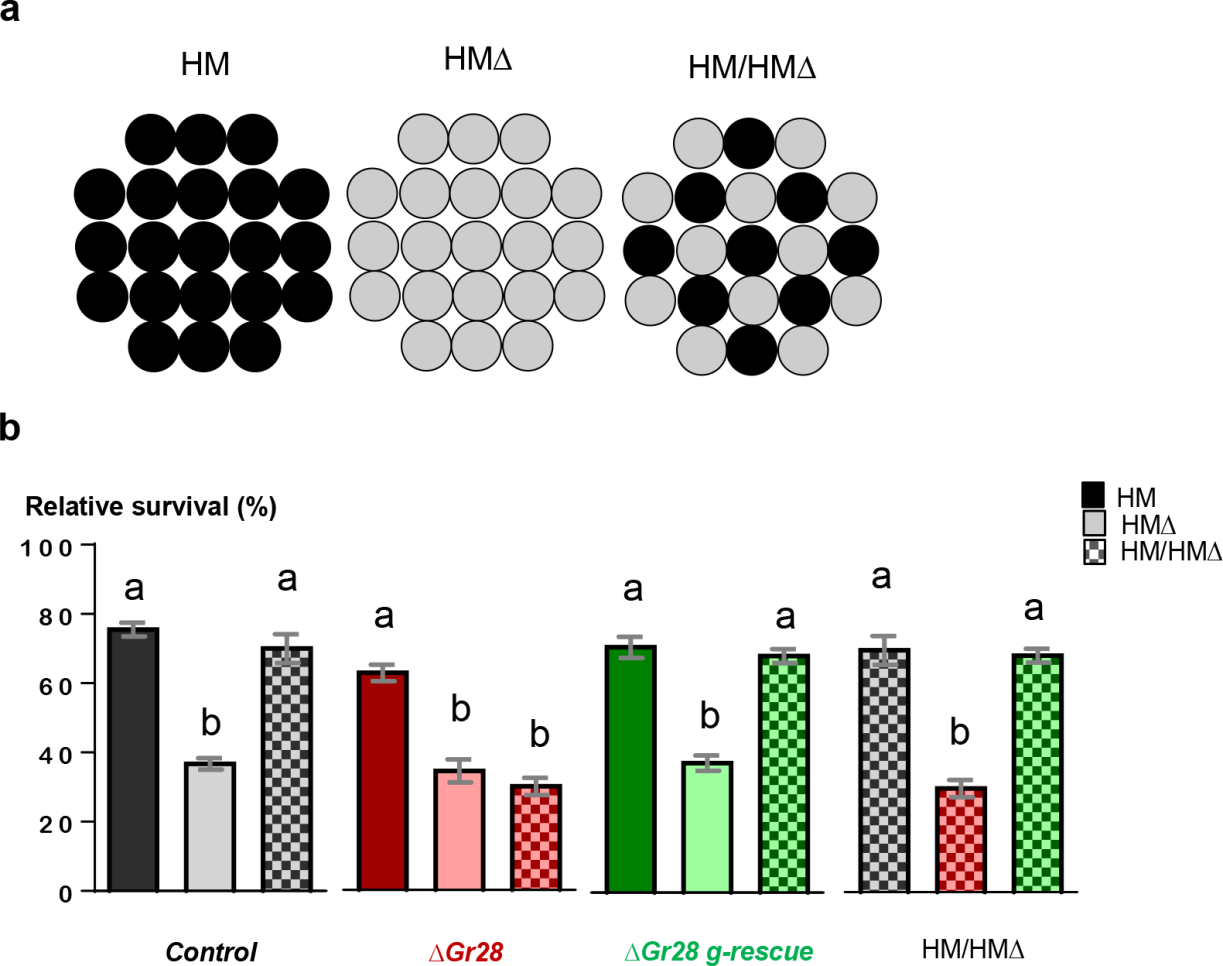
Larvae require *Gr28* genes for efficient growth and survival when presented with HM and HMΔ food. (a) About 40 eggs were deposited in 21-well microtiter plates containing 1 of 3 different foods: all wells containing HM (black; left), HMΔ (gray; middle), or a mixture of the two (12 HMΔ and 9 HM; right). Plates with either only HM or HMΔ medium were used to determine survival rate for complete (HM) or ribonucleoside-deficient (HMΔ) food. (b) Survival is displayed as percentage of flies hatched after eggs were deposited onto plate. For statistical analysis, survival in different foods was either compared across the same genotype (*Control* [black]: *w*^*1118*^, Δ*Gr28* [red]: *w*^*1118*^; Δ*Gr28/*Δ*Gr28*, *and* Δ*Gr28 g-rescue* [green]: *w*^*1118*^; Δ*Gr28/*Δ*Gr28; genGr28/+*), or different genotypes were compared against the same mixed food (light–dark checkered pattern). Each bar represents the mean ± SEM (*n* = 5–6). Bars with different letters represent significant difference (two-tailed Mann-Whitney U test, *p <* 0.05). The underlying data can be found in S4 Data. HM, holidic medium.

The experiments presented thus far identified a previously unknown taste modality for RNA (Figs 3 and 4) and 2 RNA precursors, inosine and uridine, and established a requirement for these compounds during larval life (Fig 5). Thus, we sought to determine whether the Gr28 proteins provided a competitive advantage when larvae were presented with a challenging food environment. We devised an assay in which wild-type and *Gr28* mutant larvae were required to find nucleosides in HM food during all larval life stages (Fig 6). Eggs were placed in a feeding arena that consisted of 21 wells, only 9 of which contained complete HM food while the remaining 12 contained HMΔ food (Fig 6A). Control experiments using this setup confirmed a requirement for ribonucleosides (see above), regardless of whether a *Gr28* locus was present or not (Fig 6B, compare solid versus light bars). When larvae were provided with the challenging food arena (HM/HMΔ), Δ*Gr28* mutant larvae showed a large increase in mortality (red checkered bar), while wild-type larvae or Δ*Gr28* larvae containing the *Gr28* genomic rescue construct (black and green checkered bar) showed the same high survival rate as larvae kept on HM food (solid bars). Taken together, these findings establish that larvae can discriminate between HM food based on the presence or absence of inosine and uridine and that they use this ability to increase fitness and survival when presented with a challenging food environment.

## Discussion

It has been assumed that carbohydrates, amino acids/proteins, and fats are the only major macronutrient chemicals, which serve not only as energy source but also provide the essential cellular components during growth and development. Thus, the ability to sense these 3 types of nutrient compounds by taste cells has evolved, albeit to some extent independently, in all major animal orders [1]. Here, we have established that ribonucleosides and RNA are not only potent taste ligands for larvae but also essential macronutrients necessary for the unparalleled growth and body weight gain during larval stages of *Drosophila*. Given the high level of conservation of the *Gr28* genes, it is intriguing to speculate that ribonucleosides and RNA might also be critical nutrients in other insects characterized by a rapid larval growth phase.

### The taste of ribonucleosides, a novel taste modality for fast-growing *Drosophila* larvae

We have shown that larvae sense and are attracted to ribose-containing compounds, a behavioral feature mediated by the Gr28 protein family, a set of 6 evolutionarily highly conserved Gr proteins [22]. In contrast to sugar receptors and bitter receptors [6, 7, 11, 18, 19, 31–34], the Gr28 proteins appear not to function in combination with other Gr proteins, based on multiple lines of evidence. First, many cells and neurons express only a single member of the *Gr28* gene family (Fig 2), although it remains possible that other *Gr* genes are co-expressed in some cells. Second, functional rescue experiments indicate that many Gr28 protein can restore preference for RNA and ribose in Δ*Gr28* mutant larvae when either expressed in *Gr28a* taste neurons (all 6) or in *Gr43a* fructose-sensing neurons that do not express any *Gr28* gene (3 of 6) and are therefore also unlikely to express a potential coreceptor that might be present in all *Gr28* taste neurons. However, we cannot rule out that Gr43a or another Gr present in these cells is incorporated into a Gr28-based multimeric ribose and RNA receptor complex. At the very least, our data indicate that Gr28 proteins convey specificity for these ligands. We note that a single Gr protein (Gr28b.d) conveys temperature sensing in neurons of the fly antenna [35], although in this case, no chemical ligand for this sensory modality has been identified to date. Surprisingly, while both inosine and uridine are potent ligands for Gr28, the 3 other ribonucleosides (guanosine, adenosine, and cytidine) are not. It will be interesting to determine why some, but not all, of the ribonucleosides can activate the Gr28 proteins. One possibility is that the amino group present in guanosine, adenosine, and cytidine prevents or interferes with binding of these chemicals to the Gr28 proteins.

Many *Gr28* genes are also expressed in the larval brain and the gut (Fig 2), and it is possible that these cells contribute to preference of ribose-containing substrates via a postprandial mechanism. Three lines of evidence suggest that peripheral taste is the major driver for sensing these chemicals. First, larvae respond within 2 minutes to ribose and RNA (S2 Fig). Our previous studies on the fructose receptor Gr43a have shown that such rapid decision-making is mediated by peripheral taste neurons, whereas postprandially mediated fructose sensing is a much slower process, requiring about 8 minutes to establish a clear preference [4]. Second, Gr28a-expressing taste neurons are activated by these ligands (Fig 4A), and third, some Gr28 proteins can convey ribose and RNA preference to other taste neurons (Figs 3C and 4B). Together, these observations suggest that *Gr28-*expressing taste neurons are activated by ribose and ribonucleosides and that this activation leads to the rapidly established preference for these ligands.

### Ribonucleosides and RNA are essential nutrient compounds of *Drosophila* larvae

The discovery that RNA and ribonucleosides are an essential nutrient resource recognized via a distinct taste modality represents a precedent. While detected through the ribose moiety (Figs 1, 3 and 4), our data indicate that the ribose serves only as a proxy for the detection of the nucleobase–sugar complex and not as the critical nutrient component per se, e.g., as a sugar used for energy production. In contrast to RNA, ribose complementation of HMΔ medium did not rescue development time or larval survival (Fig 5A). We propose that RNA or ribonucleosides are sensed by the larval chemosensory system because they are required in large amounts as cellular components with critical roles in gene and protein expression during the accelerated growth phase of larvae. RNA contributes about 4% and 20% of the dry weight of mammalian cells and bacteria, respectively, quantities that are in the same range as fat or polysaccharides (about 7% in both mammalian cells and bacteria). In contrast, DNA contributes only about 1% of the dry weight. Because the larval stage is characterized by unprecedented growth—evidenced by a doubling of body weight almost twice a day over a period of 4.5 days—it appears highly beneficial for larvae to be able to sense this abundant cellular constituent as an appetitive taste stimulus. In the natural environment, RNA is likely obtained from microorganisms that colonize decaying fruit.

RNAs might be of interest to chemosensory evaluation from another viewpoint. Specifically, microRNAs (miRNAs) have recently been implicated in regulating the microbiome in mammals [36], and double-stranded RNAs (dsRNAs)/small interfering RNAs (siRNAs) can cross cell membranes of the gut epithelium, a tissue well known for its ability to sense diverse types of chemicals [37]. Thus, it will be interesting to explore potential roles for Gr28 proteins in RNA sensing and transport in the larval gut, where all but 1 of the 6 genes are expressed (Fig 2).

### Diverse roles for Gr28 receptors

This paper represents the first clear evidence for a chemical compound acting as a ligand for members of this enigmatic Gr protein subfamily. While our study showed a specific role for these receptors in larval feeding on ribose-containing substrates, their function in adult flies remains to be investigated. Using proboscis extension reflex (PER) assays, we have found no evidence that adult flies respond to ribose, in either an appetitive paradigm or a feeding suppression paradigm (S3 Fig). This is not surprising given that adults, in contrast to larvae, have a much lower requirement of cell proliferation and growth, which is restricted to the female germline and stem cells in a few organs of the fly. Expression analyses have shown that the *Gr28* genes are broadly expressed in all taste organs (labial palps, tarsi and pharyngeal taste neurons), and most of them appear to be expressed in bitter taste neurons [22]. Bitter taste receptors are mutlimeric complexes that are activated by non-nutritious and often toxic chemicals and when activated suppress appetitive taste behavior [16–19]. Thus, in fly taste neurons, Gr28 subunits most likely combine with other Grs to form receptor complexes for such ligands. It will be interesting to see whether these ligands share any structural features with ribose.

Gr28 proteins have been reported to have functions in other sensory modalities, such as temperature sensing in flies and light sensing in the larvae. Specifically, Gr28b.d is expressed in 3 neurons of the aristae [22] and was later shown to be important for avoidance of warm temperatures [27]. Gr28b.d conveys thermosensitivity to a number of other cell types, suggesting that this protein acts on its own in the absence of other Gr proteins. Members of the Gr28 protein family were also implicated in light avoidance of larvae, which is mediated by multidendritic neurons in the body wall, where several of the *Gr28* genes are expressed [22] (Fig 2). The *Caenorhabditis elegans Gr28* ortholog, *lite-1*, is necessary in worms for the avoidance of visible and UV light [38–40]. Two different mechanisms have been suggested for how LITE-1 senses light: one study proposed that light avoidance is an indirect chemosensory response, related to the avoidance of hydrogen peroxide of worms [40], while another group suggested that LITE-1 directly absorbs light through 2 tryptophan residues [41]. In any case, the Gr28 proteins and its related cousin in *C*. *elegans* represent a decidedly atypical type of chemoreceptor that appears to be involved in diverse sensory modalities not associated with taste. Thus, future work will be necessary to identify additional ligands for Gr28 proteins and reveal the many roles of these receptors in physiology and behavior of insects and worms.

## Materials and methods

### Flies

Flies were raised on SCF at 25°C on a 12-hour light–dark cycle. SCF in 1.5 L of water is composed of 10.88 g of agar (*Drosophila* agar type II Genesee; 62–103), 78 g of corn meal (Genesee, 66–101), 165 g malt extract (Alternative Beverage, MUN-UL), 41.25 g of yeast extract (Genesee, 62–106), 4.69 g of propionic acid (VWR, TCP0500-500mL), 0.075 g chloramphenicol (Sigma-Aldrich, C0378), and 2.11 g of tegosept (Sigma-Aldrich, PHR1012).

### Chemicals (for two-choice feeding preference assay and imaging)

Chemicals used for two-choice feeding preference assay and CaMPARI imaging were fructose (Sigma-Aldrich, F0127), 2-deoxy-d-ribose (Sigma-Aldrich, 31170), ribose (Sigma-Aldrich, R7500), arabinose (Sigma-Aldrich, 10850), t-RNA (from brewer’s yeast; Sigma-Aldrich, 10109525001), cytidine (Sigma-Aldrich, C4654), guanosine (Sigma-Aldrich, G6752), adenosine (Sigma-Aldrich, A9251), DMSO (Sigma-Aldrich, D8418), uridine (Sigma-Aldrich, U3750), and inosine (Sigma-Aldrich, I4125).

### Larval two-choice preference assay

Third-instar feeding-stage larvae were collected from food vials by washing them out using water. They were placed along the midline of a feeding arena (plastic petri plate 60 × 15 mm, Falcon) containing freshly prepared 1% agarose on one side and 1% agarose mixed with tastant on the other side. They were left feeding, and their location (agarose versus agarose plus tastant, respectively) was recorded after 2, 4, 8, and 16 minutes. For simplicity, the preference index (PREF) was calculated for the 8-minute time point based on the number of larvae on either half of the plate. Dose-response curves for all sugars were performed using a concentration range from 25 mM to 500 mM (S2 Fig). For preference tests of wild-type and mutant larvae (Figs 1, 3, 4 and 5), a concentration of 100 mM was used for arabinose, ribose, 2-deoxyribose, and inosine, while 50 mM was used for uridine. Stock solutions for all the tastants were prepared in water before mixing in the agarose. For ribose and 2-deoxyribose, the stock solution was treated with charcoal and filtered so as to remove the unrelated odor. “PREF” indicates the number of larvae on agarose plus tastant minus the number of larvae on the agarose only, divided by the total number of larvae. A PREF score of 0 indicates no preference, while a score of +1 (or −1) indicates all larvae preferred (or avoided) tastant over agarose alone.

### Survival assay

Survival time of second-instar larvae kept on a 60 × 15 mm petri plate (Falcon) filled with 1% agarose containing various carbohydrates at 100 mM concentration and RNA at 0.5 mg/mL. Survival was monitored daily until all larvae died. Dead larvae were removed daily to avoid scavenging.

### CaMPARI calcium imaging

For calcium imaging, we used the slide preparation method as described by Alves and colleagues [42]. A single live larva was placed in 25 μl of distilled water between a cover slip and a perforated slide (a hole of 0.5 cm diameter was made on the slide with a circular drill bit). The larva was expressing CaMPARI calcium sensor described by Fosque and colleagues [29]. This preparation was placed on an inverted Nikon A1 confocal microscope and observed with 20× objective. To activate the neurons, a 25 μl solution (2× of final concentration) of the ligand was injected through the hole in the slide. After about 5 seconds, a pulse of photoconversion (PC) light of 405 nm with a power of approximately 1.8 w/cm^2^ was delivered to the larvae for 10 seconds. Post activation, the neurons were observed for conversion from green to red emitted wavelengths. The changes were calculated as the ratio of red/green fluorescence (F_RED_/F_GREEN_). Data were acquired using the Nikon NIS element acquisition and analysis package. Data are expressed as mean ± SEM. To determine preexposure values, the images were taken without applying ligand or PC light. To determine PC-light–only values, images were taken with applying PC light but without applying ligand.

### HM

HM was prepared based on diet reported by Piper and colleagues [30]. A detailed list of chemicals is listed in S1 Table. For HMΔ food, inosine and uridine were removed. For supplementation experiments, HMΔ food was complemented by addition of RNA and ribose final concentrations of 0.5 mg/mL and 0.5 mM, respectively. For survival experiments, HM food and its derivatives were presented in 60 × 15 mm plastic petri plates (Falcon 5 ml/plate; Fig 5) or in 21-well microtiter plates (350 μl/well; Fig 6). These plates were then embedded in a 60 × 15 petri plate using 3% agarose. After plates were at room temperature, 40 eggs were placed on each plate, which were sealed with a perforated lid and placed in a humid chamber maintained at 25°C in a 12-hour light–dark cycle. Larvae location was checked once per day, and dead larvae were removed to avoid scavenging.

### Statistical analysis

In all figure legends, *n* indicates the number of experiments, unless otherwise noted. For each experiment, data are presented as mean ± SEM. Statistical analysis was performed using two-tailed nonparametric Mann-Whitney U test to compare 2 different groups of samples. *p* < 0.05 was considered to be statistically significant. Statistical analyses were conducted using Prism 6.0 software (GraphPad Software).

### Proboscis Extension Reflex (PER) assay

PER assays were carried out as described in Slone and colleagues [7] with the following modifications. Male and female flies were collected on the day of eclosion and kept on standard corn meal food for 5 to 6 days at 25°C. Prior to performing PER assays, flies were starved for 24 to 26 hours at 25°C in empty vials with a water-saturated cotton ball. Flies were immobilized by cooling briefly on ice and wing-mounted dorsally on a microscope slide using double-sided Scotch tape. Legs were taped to the slide. Mounted flies were allowed to recover for 60 to 90 minutes at room temperature in a humidified chamber. Flies were then allowed to drink water until satiation to ensure that PER responses were nutrient derived. Flies showing no response to water were excluded. Each fly was tested with a given tastant by briefly applying the taste solution to the labellum. Each fly was tested 3 times for each taste solution. A PER response was scored as positive (1) if the proboscis was fully extended, otherwise it was scored as negative (0). PER response scores (%) from a single fly were 0% (0/3 responses in the 3 applications), 33% (1/3), 66% (2/3), or 100% (3/3). Flies were allowed to drink water after each taste application. Taste solutions were delivered with a 20 ml pipette. Stock solutions of sucrose (Macron, Cat No. 8360–06) and ribose (Sigma, Cat No. R7500) were prepared in Millipore Q water and kept at 5°C. Stock solutions were diluted to the final concentration using Millipore Q water prior to each experiment.

## Supporting information

S1 Table HM chemical composition. The HM used in this study consisted of the chemicals listed in the table. Final concentration in the medium is indicated. Preparation was carried out as described by Piper and colleagues [1]. aa: amino acid; ess aa: essential amino acid; HM, holidic medium; non ess aa: nonessential amino acid.
(DOCX)

S1 Fig Gr28a and Gr43a are expressed in different larval taste neurons. *Gr28a-GAL4* is expressed in a pair of terminal taste neurons on each side of the tip of the head (left), while *Gr43a*^*GAL4*^ is expressed in a pair of neurons associated with the dorsal and ventral pharyngeal taste organ (middle). The different location of these taste neurons is revealed clearly when the 2 drivers are combined in the same larvae (middle). Genotypes: *w*^*1118*^*; Gr28a-GAL4/UAS mCD8*:*GFP* (left), *w*^*1118*^*; Gr43a*^*GAL4*^*/UAS mCD8*:*GFP* (middle), *w*^*1118*^*; Gr28a-GAL4 Gr43a*^*GAL4*^*/UAS mCD8*:*GFP* (right). Scale bar is 100 μm.
(TIF)

S2 Fig Two-choice preference assay of larvae using a range of concentrations to various ligands over time. Agarose plates were prepared with different concentration of indicated ligand on one side and agarose on the opposite site. Location of *w*^*1118*^ larvae was recorded at 2, 4, 8, and 16 minutes, and PREF was calculated as described in Materials and methods. Each line represents the mean ± SEM (*n* = 6–30). The underlying data can be found in S5 Data. PREF, preference index.
(TIF)

S3 Fig Adult flies do not respond to ribose. (a) Flies readily responded to 100 mM sucrose but not to ribose, even at the highest concentration (800 mM), indicating that they lack intrinsic appetitive ribose taste. (b) Addition of ribose 100 mM sucrose does not reduce PER, indicating that ribose is not a repulsive stimulus, unlike bitter compounds. Numbers in parenthesis indicates mM concentrations of sucrose and ribose. The underlying data can be found in S5 Data.
(TIF)

S1 Data (XLSX)

S2 Data (XLSX)

S3 Data (XLSX)

S4 Data (XLSX)

S5 Data (XLSX)

## Abbreviations

CaMPARI: Calcium Modulated Photoactivatable Ratiometric Integrator
CNS: central nervous system
DPS: dorsal pharyngeal sensory
dsRNA: double-stranded RNA
GFP: green fluorescent protein
Gr: gustatory receptor
Gr28: gustatory receptor subfamily 28
HM: holidic medium
IR: Ionotropic receptor
miRNA: microRNA
PC: photoconversion
PER: proboscis extension reflex
PPS: posterior pharyngeal sensory
PREF: preference index
SCF: standard cornmeal food
siRNA: small interfering RNA
TO: terminal organ
UV: ultraviolet
VPS: ventral pharyngeal sensory
