# Supplementary material for: The taste of ribonucleosides: Novel macronutrients essential for larval growth are sensed by Drosophila gustatory receptor proteins
Source: PLoS Biol. 2018 Aug 7;16(8):e2005570. doi: 10.1371/journal.pbio.2005570 (PMC6080749; doi:10.1371/journal.pbio.2005570)
Supplement: S1 Table — The HM used in this study consisted of the chemicals listed in the table. Final concentration in the medium is indicated. Preparation was carried out as described by Piper and colleagues [1]. aa: amino acid; ess aa: essential amino acid; HM, holidic medium; non ess aa: nonessential amino acid. (DOCX) [file pbio.2005570.s001.docx]

| \| **Chemical compound** \| **Final concentration** \| **w/v (g/L)** \| **Source** \| \| --- \| --- \| --- \| --- \| \| cholesterol \|  \| 1 \| Sigma C8667 \| \| agar \| 2% \| 2% \| Difco 214530 \| \| L-isoleucine \| 8.84 mM \| 1.16 \| Sigma I2752 \| \| L-leucine \| 12.5 mM \| 1.64 \| Sigma L8912 \| \| L-tyrosine \| 4.64 mM \| 0.84 \| Sigma T3754 \| \| sucrose \| 50 mM \| 17.12 \| Macron CAS#57-50-1 \| \|  \|  \|  \|  \| \| **trace element** \|  \|  \|  \| \| CaCl2.6H2O \| 1.14 mM \| 0.25 \| EMD CAS#10035-04-8 \| \| MgSO4 \| 2.08 mM \| 0.25 \| Sigma M7506 \| \| CuSO4.5H2O \| 10.01µM \| 0.0025 \| Sigma C7631 \| \| FeSO4.7H2O \| 89.93 µM \| 0.025 \| Sigma F7002 \| \| MnCl2.4H2O \| 5.05 µM \| 0.001 \| Sigma M3634 \| \| ZnSO4.7H2O \| 86.94 µM \| 0.025 \| Sigma Z0251 \| \| boil \| \| \| \| \| **acetate buffer** \|  \|  \|  \| \| glacial acetic acid \| 52.46 mM \| 0.30% \| EMD AX0073-6 \| \| KH2PO4 \| 22.06 mM \| 3 \| EMD PX1561-1 \| \| NaHCO3 \| 11.90 mM \| 1 \| EMD SX0320-1 \| \|  \|  \|  \|  \| \| **lipid-related** \|  \|  \|  \| \| myo-inositol \| 27.98 µM \| 0.0050 \| Sigma I7508 \| \| choline- chloride \| 358.11 µM \| 0.050 \| Sigma C1879 \| \| uridine \| 245.70 µM \| 0.065 \| Sigma U3750 \| \| inosine \| 242.48 µM \| 0.060 \| Sigma I4125 \| \|  \|  \|  \|  \| \| **aa solution** \|  \|  \|  \| \| **ess aa** \|  \|  \|  \| \| phenylalanine \| 5.55 mM \| 0.92 \| Sigma P2126 \| \| histidine \| 4.38 mM \| 0.68 \| Sigma H8000 \| \| lysine \| 11.90 mM \| 1.74 \| Sigma L5626 \| \| methionine \| 2.28 mM \| 0.34 \| Sigma M9625 \| \| arginine \| 8.15 mM \| 1.42 \| Sigma A5131 \| \| threonine \| 10.83 mM \| 1.29 \| Sigma T8625 \| \| valine \| 11.44 mM \| 1.34 \| Sigma V0500 \| \| tryptophan \| 2.15 mM \| 0.44 \| Sigma T9753 \| \|  \|  \|  \|  \| \| **non ess aa** \|  \|  \|  \| \| alanine \| 17.82 mM \| 1.59 \| Sigma A7627 \| \| aspartic acid \| 6.32 mM \| 0.84 \| Sigma A6683 \| \| glycine \| 14.42 mM \| 1.08 \| Biosciences Cat#RC-054 \| \| asparagine \| 6.36 mM \| 0.84 \| Sigma A0884 \| \| proline \| 4.89 mM \| 0.56 \| Sigma P0380 \| \| glutamine \| 10.87 mM \| 1.82 \| Sigma G3126 \| \| serine \| 7.22 mM \| 0.76 \| Sigma S4500 \| \| cystein \| 2.18 mM \| 0.26 \| Sigma C7352 \| \| sodium glutamate \| 10.76 mM \| 1.82 \| Sigma G5889 \| \|  \|  \|  \|  \| \| **vitamin solution** \|  \|  \|  \| \| thiamine \| 5.28 µM \| 0.0014 \| Sigma T4625 \| \| riboflavine \| 1.86 µM \| 0.00070 \| Sigma R7649 \| \| nicotinic acid \| 68.23 µM \| 0.0084 \| Sigma N4126 \| \| Ca pantotherate \| 50.18 µM \| 0.011 \| Sigma 21210 \| \| pyridoxine \| 10.33 µM \| 0.0017 \| Sigma P9755 \| \| biotin \| 0.57 µM \| 0.00014 \| Sigma B4501 \| \|  \|  \|  \|  \| \| folic acid \| 1.13 µM \| 0.0005 \| Sigma F7876 \| \|  \|  \|  \|  \| \| propionic acid \| 80 mM \| 0.60% \| J.T. Baker U330-09 \| \| tegocept \| 98.6 mM \| 1.5 \| Apex Cat#20-259 \|   **S1 Table** |
| --- | --- | --- | --- | --- | --- | --- | --- | --- | --- | --- | --- | --- | --- | --- | --- | --- | --- | --- | --- | --- | --- | --- | --- | --- | --- | --- | --- | --- | --- | --- | --- | --- | --- | --- | --- | --- | --- | --- | --- | --- | --- | --- | --- | --- | --- | --- | --- | --- | --- | --- | --- | --- | --- | --- | --- | --- | --- | --- | --- | --- | --- | --- | --- | --- | --- | --- | --- | --- | --- | --- | --- | --- | --- | --- | --- | --- | --- | --- | --- | --- | --- | --- | --- | --- | --- | --- | --- | --- | --- | --- | --- | --- | --- | --- | --- | --- | --- | --- | --- | --- | --- | --- | --- | --- | --- | --- | --- | --- | --- | --- | --- | --- | --- | --- | --- | --- | --- | --- | --- | --- | --- | --- | --- | --- | --- | --- | --- | --- | --- | --- | --- | --- | --- | --- | --- | --- | --- | --- | --- | --- | --- | --- | --- | --- | --- | --- | --- | --- | --- | --- | --- | --- | --- | --- | --- | --- | --- | --- | --- | --- | --- | --- | --- | --- | --- | --- | --- | --- | --- | --- | --- | --- | --- | --- | --- | --- | --- | --- | --- | --- | --- | --- | --- | --- | --- | --- | --- | --- | --- | --- | --- | --- | --- | --- | --- | --- | --- | --- | --- | --- | --- | --- | --- | --- | --- | --- | --- | --- | --- | --- | --- | --- | --- | --- | --- | --- | --- | --- | --- | --- | --- | --- | --- | --- | --- | --- | --- | --- | --- | --- | --- | --- | --- | --- | --- | --- | --- | --- | --- | --- | --- | --- | --- | --- |
|  |
| REFERENCE  1. Piper MD, Blanc E, Leitao-Goncalves R, Yang M, He X, Linford NJ, et al. A holidic medium for Drosophila melanogaster. Nature methods. 2014;11(1):100-5. doi: 10.1038/nmeth.2731. PubMed PMID: 24240321; PubMed Central PMCID: PMCPMC3877687. |
