# Supplementary figures and images for: The taste of ribonucleosides: Novel macronutrients essential for larval growth are sensed by Drosophila gustatory receptor proteins
Source: PLoS Biol. 2018 Aug 7;16(8):e2005570. doi: 10.1371/journal.pbio.2005570 (PMC6080749; doi:10.1371/journal.pbio.2005570)

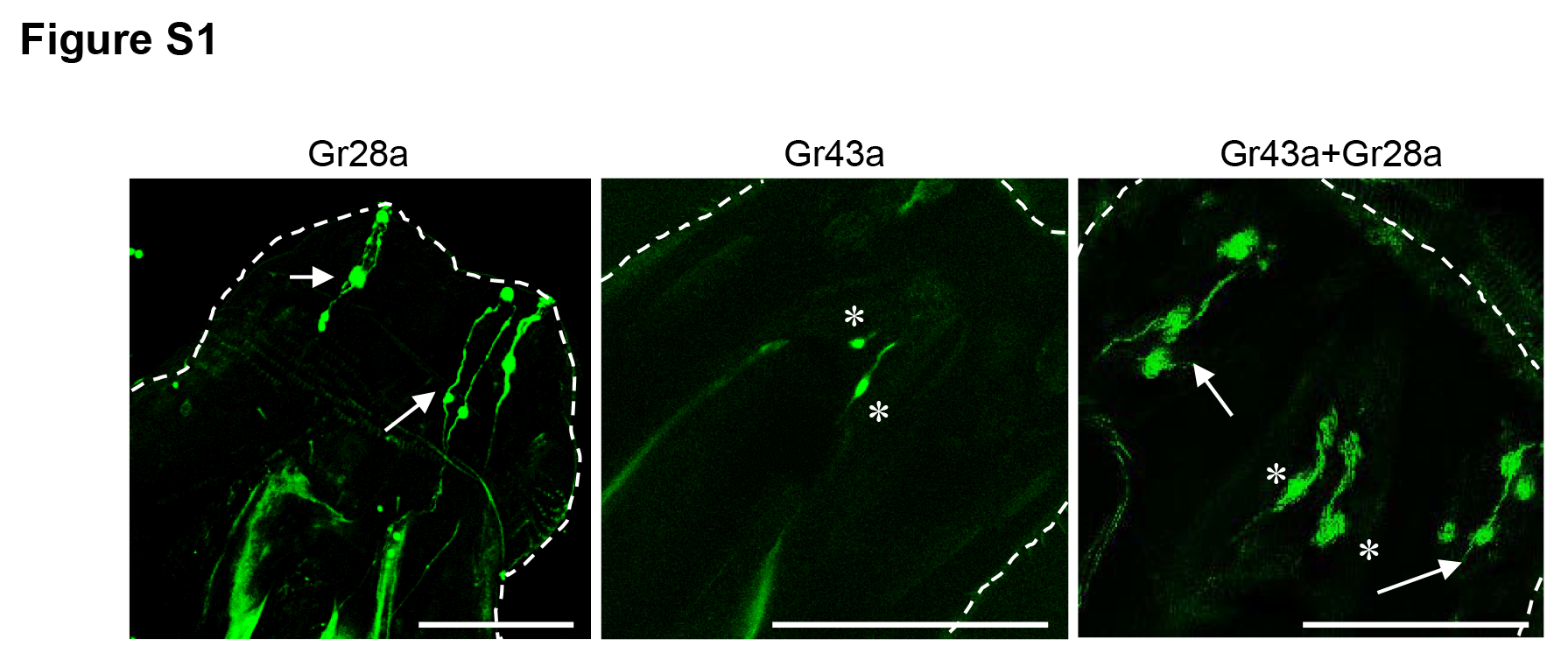

Supplement: S1 Fig — Gr28a-GAL4 is expressed in a pair of terminal taste neurons on each side of the tip of the head (left), while Gr43aGAL4 is expressed in a pair of neurons associated with the dorsal and ventral pharyngeal taste organ (middle). The different location of these taste neurons is revealed clearly when the 2 drivers are combined in the same larvae (middle). Genotypes: w1118; Gr28a-GAL4/UAS mCD8:GFP (left), w1118; Gr43aGAL4/UAS mCD8:GFP (middle), w1118; Gr28a-GAL4 Gr43aGAL4/UAS mCD8:GFP (right). Scale bar is 100 μm. (TIF) [file pbio.2005570.s002.tif]

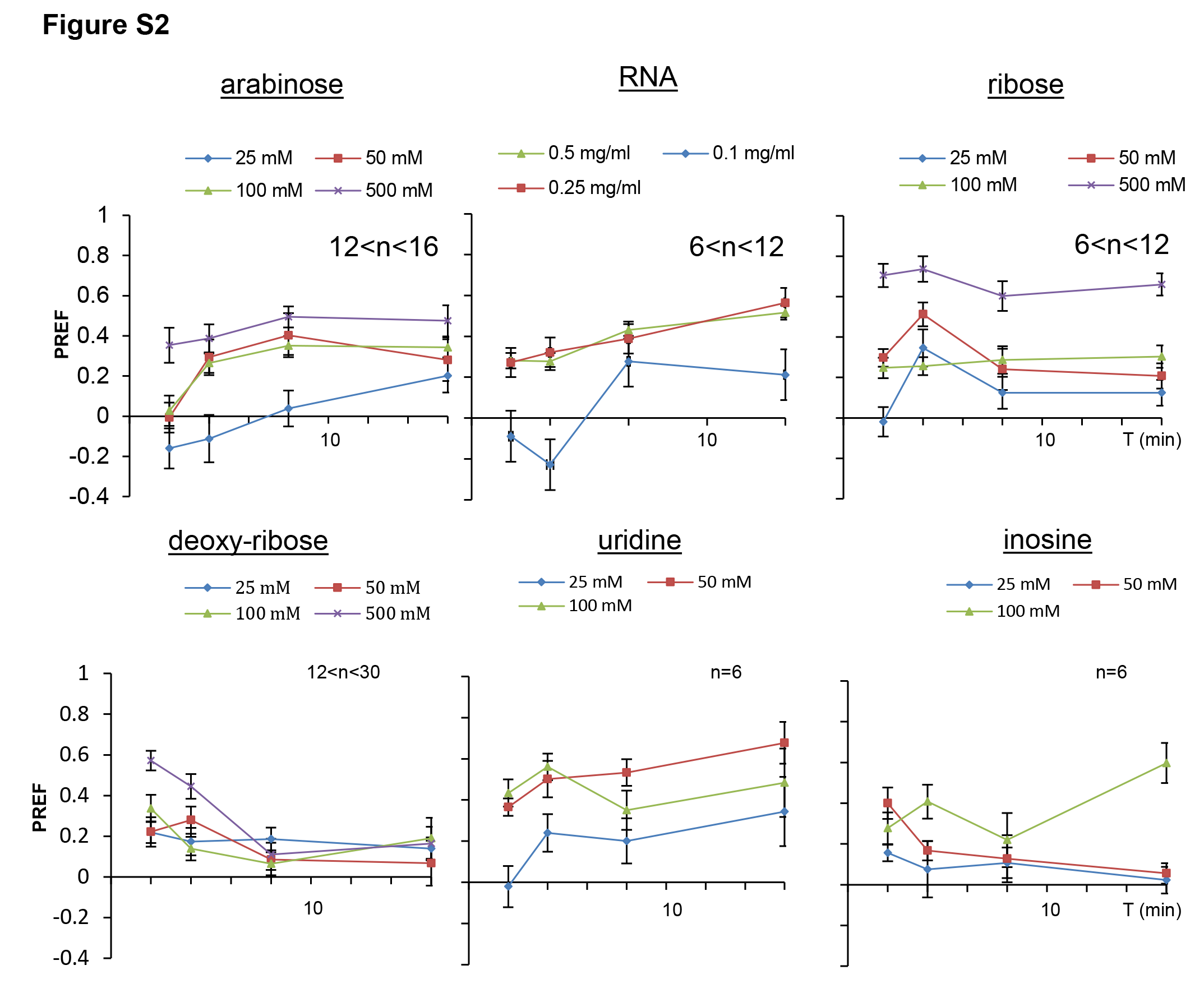

Supplement: S2 Fig — Agarose plates were prepared with different concentration of indicated ligand on one side and agarose on the opposite site. Location of w1118 larvae was recorded at 2, 4, 8, and 16 minutes, and PREF was calculated as described in Materials and methods. Each line represents the mean ± SEM (n = 6–30). The underlying data can be found in S5 Data. PREF, preference index. (TIF) [file pbio.2005570.s003.tif]

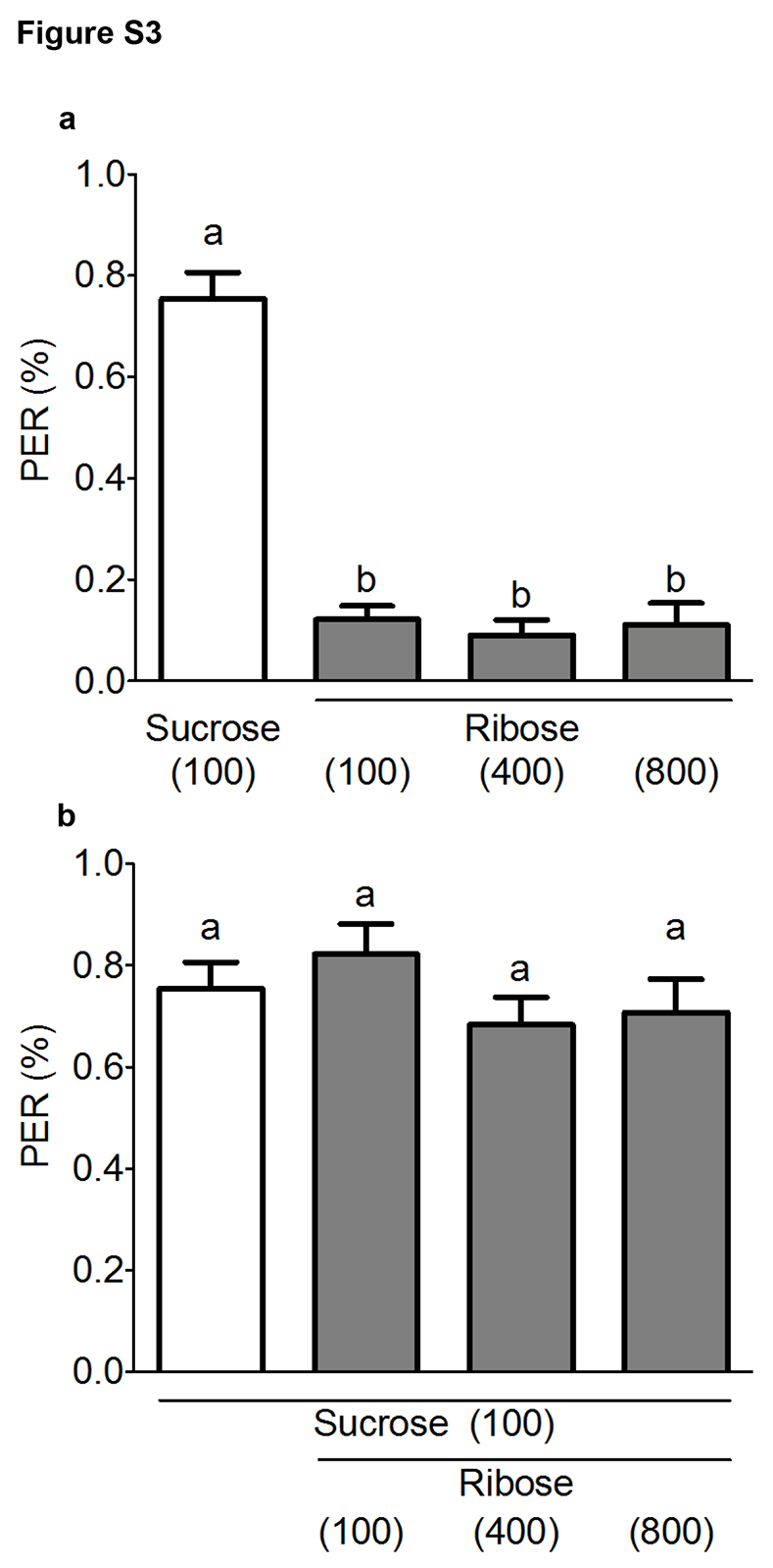

Supplement: S3 Fig — (a) Flies readily responded to 100 mM sucrose but not to ribose, even at the highest concentration (800 mM), indicating that they lack intrinsic appetitive ribose taste. (b) Addition of ribose 100 mM sucrose does not reduce PER, indicating that ribose is not a repulsive stimulus, unlike bitter compounds. Numbers in parenthesis indicates mM concentrations of sucrose and ribose. The underlying data can be found in S5 Data. (TIF) [file pbio.2005570.s004.tif]
